# A Case Report: Co-presenting COVID-19 Infection and Acute Drug Intoxication

**DOI:** 10.5811/cpcem.2020.6.47764

**Published:** 2020-07-01

**Authors:** Jeremy Riekena, Irene Lee, Anita Lui, Marion-Vincent Mempin

**Affiliations:** New York-Presbyterian Queens, Department of Emergency Medicine, Flushing, New York

**Keywords:** COVID-19, Anchoring bias, Opiates

## Abstract

**Background:**

Coronavirus disease 2019 (COVID-19) has spread throughout the world since late 2019. Symptoms appear after a two-week incubation period and commonly include fever, cough, myalgia or fatigue, and shortness of breath.

**Case Report:**

A 32-year-old male with a history of opiate abuse presented to the emergency department with altered mental status. The patient was lethargic and hypoxic with improvement from naloxone. Official chest radiograph was read as normal; however, the treating clinicians noted bilateral interstitial opacities, raising concern for underlying infectious etiology. Opiates and cocaine were positive on drug screen, and an arterial blood gas on room air showed hypoxemia with respiratory acidosis. The patient was intubated during the treatment course due to persistent hypoxemia and for airway protection after resuscitation. The COVID-19 test was positive on admission, and later computed tomography showed ground-glass opacities. The patient was extubated and discharged after one week on the ventilator.

**Conclusion:**

When screening patients at and during evaluation, physicans should consider a broad differential as patients with atypical presentations may be overlooked as candidates for COVID-19 testing. As screening and evaluation protocols evolve, we emphasize maintaining a high index of suspicion for COVID-19 in patients with atypical symptoms or presenting with other chief complaints in order to avoid spreading the disease.

## INTRODUCTION

The main objective in this case study was to promote a bottoms-up approach in tackling the coronavirus disease 2019 (COVID-19) pandemic, as it has a wide variety of presentations in different individuals across the globe. Throughout this case, we were able to use multiple points of re-evaluation to uncover COVID-19 induced hypoxemia initially presenting as an opiate overdose.

In December 2019, a cluster of patients who had been admitted to various hospitals in the eastern region of China were diagnosed with pneumonia of unknown etiology. After undergoing a series of medical evaluations, it was concluded that these individuals were epidemiologically linked to a seafood and wet-animal wholesale market located in Wuhan, Hubei Province, China. Upon further review, it was determined that these patients had been infected with a novel variation of coronavirus.^1^

Coronavirus is a major pathogen that predominantly targets the human respiratory system. Symptoms of this virus, on average, appear after an incubation period of approximately 5.2 days. Onset of symptoms to death varies between 6–41 days, with a median onset period of 14 days. Mortality is predicated on various risk factors including the patient’s age and comorbidities.

At the onset of illness in the setting of the COVID-19 pandemic, most common presentations in patients include fever (77–98%), cough (46–82%), myalgia or fatigue (11–52%), and shortness of breath (3–31%).^2^ However, clinicians should not neglect other atypical symptoms that have been reported, including sore throat, headache, hemoptysis, diarrhea, and nausea. Research done by Xu et al showed that pathological features of COVID-19 greatly resemble those seen in severe acute respiratory syndrome (SARS)-associated coronavirus, as well as Middle Eastern respiratory syndrome coronavirus infection. Methods to identify various modes of transmission are crucial in the development of transmission mitigation strategies and creation of therapeutics to more effectively manage the disease.

Lab values that help identify COVID-19 have included leukopenia and lymphopenia, aspartate aminotransferase (AST) and alanine aminotransferase (ALT) elevation, increase in acute inflammatory markers, and low procalcitonin.^3^ Imaging findings include chest radiograph (CXR) with bilateral interstitial opacities. Numerous peripheral ground-glass opacities have been observed in subpleural areas of both lungs on computed tomography (CT), mediated by both general and localized immune responses that led to inflammation within the lungs. A limited number of reports describe identification of hypoxic COVID-19 patients with an absence of the most common respiratory or systemic symptoms.

## CASE REPORT

A 32-year-old male presented to the emergency department (ED) with altered mental status secondary to drug overdose as reported by emergency medical services (EMS). EMS gave the patient one round of naloxone with reported improvement of the patient’s respiratory status. Patient chart review was notable for a history of opiate dependence and enrollment in suboxone and methadone centers. History of presenting illness was limited due to the patient’s presenting mental status.

Presenting vital signs were notable for a respiratory rate of 25 breaths per minute and oxygen saturation of 75% on room air, with improvement to 95% on a non-rebreather mask. The patient was lethargic but arousable and was able to move all four extremities spontaneously. Initial lab work was notable for a glucose of 87 milligrams per deciliter (mg/dL) (70–130 mg/dL) and a urine drug screen that was positive for opiates and cocaine. The official CXR read was as normal; however, the treating clinicians were concerned about the subtle appearance of bilateral interstitial opacities.

After a period of observation, the patient had multiple episodes of emesis. Additionally, he was noted to have no improvement in mental status or ability to oxygenate; therefore, crystalloid fluids, naloxone, ondansetron, and clonidine were given in an attempt to reverse the reported opiate overdose and prevent withdrawal symptoms. On re-assessment, physical exam revealed sonorous respiration and inspiratory stridor; thus, it became more apparent that an underlying pulmonary and metabolic pathology was contributing to the patient’s hypoxemia and lethargy, rather than just opiate use.

**CPC-EM Capsule**

What do we already know about this clinical entity?

Coronavirsus disease 2019 (COVID-19) is a world wide pathogen with a varied symptom course. Major health organizations recommend measures to prevent spread between asymptomatic individuals.

What makes this presentation of disease reportable?

Early in the COVID-19 pandemic and before routine testing, this patient was identified with COVID-19 infection in addition to opiate overdose with hypoxia.

What is the major learning point?

As the COVID-19 pandemic wanes, maintaining a high index of suspicion for asymptomatic or occult infection is important in disease control.

How might this improve emergency medicine practice?

This case highlights the importance of continuous re-evaluation for multiple disease processes underlying a pre-established diagnosis.

Once the patient’s family arrived at the hospital, they noted that he had recently traveled to Israel for drug rehabilitation approximately one week prior to his hospital visit. However, they believed that he continued to use intravenous drugs upon arrival home. At that time, Israel already had confirmed COVID-19 positive cases.^4^

The patient was moved into a negative pressure isolation after travel history correlated with the known abnormal CXR and hypoxemia because of a high degree of suspicion of COVID-19. Arterial blood gas on room air showed a pH of 7.26 (7.35–7.45), partial pressure carbon dioxide of 60 millimeters of mercury (mmHg) (35–45 mmHg), partial pressure oxygen of 47mmHg (80–100 mmHg), and bicarbonate of 27 milliequivalents per liter (mEq/L) (22–28 mEq/L), demonstrating hypercarbic hypoxemic respiratory failure, and the patient’s oxygen saturation began to drop to 85% on a non-rebreather.

**Image 1:**
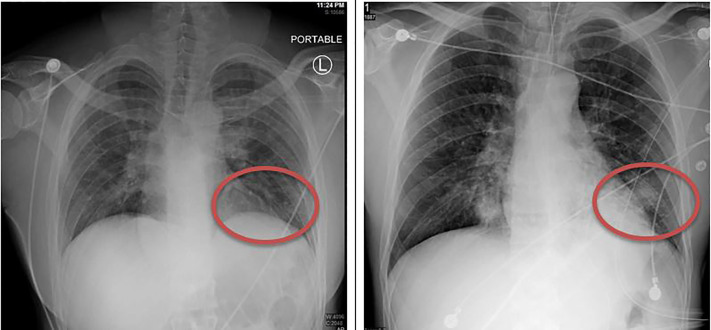
Initial chest radiograph (left) and post-intubation chest radiograph (right) demonstrating interval progression of bibasilar opacities and interstitial opacities in several hours.

**Image 2:**
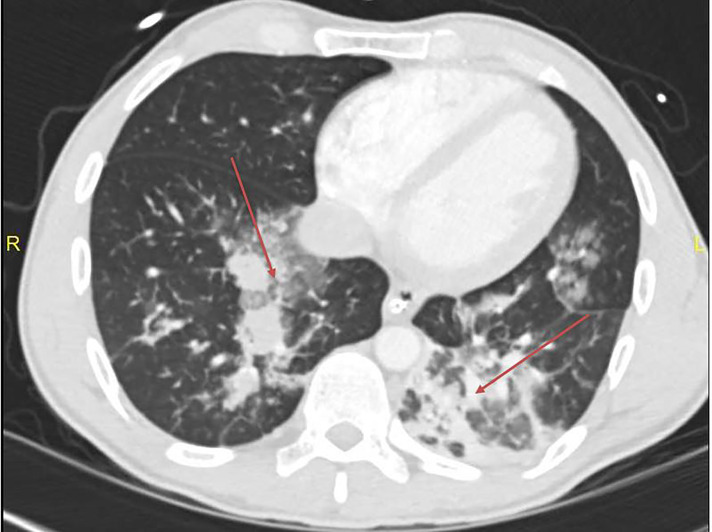
Computed tomography showing extensive ground-glass opacities (arrows) in multiple lung fields concerning for inflammatory or infectious etiology.

The decision was made to intubate the patient for hypoxemia and airway protection. A post-intubation CXR demonstrated progression of bibasilar opacities from initial CXR (Image 1), with later lung CT demonstrating extensive ground-glass opacities in bilateral lungs (Image 2).

During the patient’s intensive care unit (ICU) course, the SARS-associated coronavirus ribonucleic acid assay sent from the ED came back positive, indicating COVID-19 pulmonary infection. Repeat lab work also showed characteristic COVID-19 findings of lymphopenia and elevated AST and ALT. After six days on a ventilator, the patient was successfully extubated. He was treated with hydroxychloroquine for COVID-19 and antibiotics for aspiration pneumonia. After a stable course on the general medical floor, he was discharged home with self-quarantining instructions to prevent the spread of COVID-19.

## DISCUSSION

Anchoring bias is a common phenomenon in the field of medicine, and especially in emergency medicine. In the acute setting in the ED, a patient’s history and ancillary information may be limited due to various reasons, such as the altered mental status presented in our patient. Because of these limitations, it is critical to maintain a broad and evolving differential diagnosis using thorough physical examinations and continuous re-evaluations.

Our 32-year-old patient presented as a drug overdose with agitation and was ultimately admitted to the medical ICU after intubation for profound hypoxemia and airway protection. While we were initially limited to information given by EMS, the lab results and imaging provided us with information that was compatible with patients who had tested positive for COVID-19. Variables such as oxygen saturation and abnormal CXR findings were the initial clues into the presenting viral illness. The fact that the patient’s mental status did not improve, paired with the ancillary history after family arrived in the ED, made it clear that COVID-19 had to be considered underlying the patient’s presentation. After definitive airway control and isolation, a contrast-enhanced CT of the lungs was performed, showing the ground-glass opacities prevalent with COVID-19 positive patients, indicating that the patient was an appropriate candidate for COVID-19 testing. COVID-19 primarily affects the respiratory system, thus directing the clinicians’ focus to respiratory-related symptoms.

When screening patients at and during evaluation, clinicians should consider a broad differential because patients with atypical presentations may be overlooked as candidates for COVID-19 testing and treatment. Ramifications of neglecting certain signs and symptoms may include risks such as patients being brought to non-COVID-19 isolation areas and causing further spread of the disease to otherwise healthy individuals. By thoroughly considering all potential risk factors within variable presentations, the capture rate of COVID-19 infected patients should improve. Further discussion and studies should be encouraged as the rapid rise in COVID-19 patients may precipitate mistakes made while they are being triaged. Given the paucity of research on atypical presentations for COVID-19, it would prove beneficial to broaden the knowledge of this pandemic.

Hospital protocols are rapidly developing and evolving to account for the wide variety of patient presentations. These include patients with absence of all respiratory symptoms; incidental findings on CT or CXR while investigating other pathology; and exacerbation of chronic disease such as hyperglycemia in diabetic patients. No protocol, however, can effectively capture all patients who may fall out of normal protocol guidelines. In an effort to curb the spread of disease, physicians will need a high index of suspicion for identifying COVID-19 positive patients, especially as the incidence of the disease continues to rise.

## CONCLUSION

Keeping a broad differential diagnosis while evaluating patients during the COVID-19 pandemic will aid physicians in controlling the spread of this pathogen. Appropriate use of imaging and labs may expedite diagnosis of COVID-19 in patients and ensure they are properly managed. Atypical manifestations of patients with COVID-19 should not be undermined as research is still limited on the pathophysiology of this virus.
